# Letter on “Bioimpedance-assessed muscle wasting and its relation to nutritional intake during the first week of ICU: a pre-planned secondary analysis of Nutriti Study”

**DOI:** 10.1186/s13613-024-01272-8

**Published:** 2024-03-07

**Authors:** Libing Jiang, Shanxiang Xu

**Affiliations:** Department of Emergency Medicine, Second Affiliated Hospital, Zhejiang University School of Medicine, Hangzhou, China; Key Laboratory of The Diagnosis and Treatment of Severe Trauma and Burn of Zhejiang Province, Hangzhou, China; Zhejiang Province Clinical Research Center for Emergency and Critical Care Medicine, Hangzhou, 310009 China

Dear editor

We read with great interest the article by Cristian et al. entitled “Bioimpedance-assessed muscle wasting and its relation to nutritional intake during the first week of ICU: a pre-planned secondary analysis of Nutriti Study” published in Annals of Intensive Care [1].

The authors evaluated muscle mass changes with bioimpedance analysis(BA) during the first 7 days after intensive care unit (ICU) admission. Meanwhile they explored the correlations between muscular loss(MM) and caloric and protein debt. We would like to congratulate the authors for the considerable efforts they made to explore the evalue of BA in evaluating MM and guiding nutrtion treatment in critically ill paltients. However, a few reservations are worth considering.

First, the statistical analysis used in this study needs further explanation. “Continuous variables are reported as median (interquartile range [IQR]) or mean (SD) as appropriate” the authors described in the methods section. Either MM or phase angle were reported as median (interquartile range [IQR]) in Table 3, indicating the data may not meet the characteristics of a normal distribution. However, one of the prerequisites for the T-test is the datas should be (approximately) normally distributed. Thus, T-tests were used to compare MM and phase angle at different time points may not be appropriate. In terms of data presentation alone, non-parametric tests may be more appropriate. Meanwhile, another prerequisites for the T-test is the datas should be independent. As we all konw, the design of this study was repeated measures, and the individual data is dependent. This also chanllenge the choice of T test. Combined with the characteristics of the data and the design of the study, single-factor repeated measures of analysis of variance or more advanced statistical methods may be more appropriate. In addtion, correct sample size is important in clinical research, so we wonder whether the sample size of this study is sufficient to support the research conclusions.

Second, the European Society of Parenteral and Enteral Nutrition’s (ESPEN) 2018 critical care nutrition guideline introduces stages of critical illness in making nutrition recommendations [2]. The first week of critical illness is the acute phase and further divided into early (days 1–2) and late acute phase (days 3–7). The time-points are arbitrary, and uptonow an objective marker to distinguish phases does not exist. During the early acute phase the patients just suffer a severe injury, the body enters a catabolic state. Catabolism and anabolic resistance will occur whether or not exogenous nutrition is provided. The above mentioned characteristics in the early acute phase theoretically support the results obseved in the present study that the total amount of calories and proteins does not correlate with changes in MM and phase angle. The late acute phase is a transitional period, during which recovery will begin, and catabolism will wane. Nutritional administration can be ramped up during this period. The total amount of calories and proteins may be positive correlate with changes in MM and phase angle during this phase. Thus it would be better if the authors explore the relationships bettween the total amount of calories and proteins and changes in MM and phase angle according to different stages of critical illness.

Finally, 83% of included patients in this study received vasopressors. The use of vasopressors is one of important factors we should consider in making feeding strategy, especially the dose of vasoactive drugs [3]. The application of full dose feeding in patients receiving high dose of vasopressors did not result in better prognosis and was associated with a higher rate of complications [4]. And early calorie and protein restriction was associated with faster recovery and fewer complications [5]. The detailed doses of vasoactive drugs were not decribed in the present study, and this may bias the results. It would be better to explore the difference of results according to the patient whether received vasopressors.

## Data Availability

My manuscript has no associated data.

## Abbreviations

BA: Bioimpedance analysis
ICU: Intensive care unit
MM: Muscular loss
IQR: Interquartile range
SD: Standard deviation
ESPEN: European Society of Parenteral and Enteral Nutrition
